# Exploring the binding of rationally engineered tandem-repeat proteins to E3 ubiquitin ligase Keap1

**DOI:** 10.1093/protein/gzab027

**Published:** 2021-12-09

**Authors:** Sarah K Madden, Laura S Itzhaki

**Affiliations:** Department of Pharmacology, University of Cambridge, Cambridge CB2 1PD, UK; Department of Pharmacology, University of Cambridge, Cambridge CB2 1PD, UK

**Keywords:** biologics, Nrf2, peptide grafting, tandem-repeat protein, tetratricopeptide repeat

## Abstract

The process of displaying functional peptides by ‘grafting’ them onto loops of a stable protein scaffold can be used to impart binding affinity for a target, but it can be difficult to predict the affinity of the grafted peptide and the effect of grafting on scaffold stability. In this study, we show that a series of peptides that bind to the E3 ubiquitin ligase Keap1 can be grafted into the inter-repeat loop of a consensus-designed tetratricopeptide repeat (CTPR) protein resulting in proteins with high stability. We found that these CTPR-grafted peptides had similar affinities to their free peptide counterparts and achieved a low nanomolar range. This result is likely due to a good structural match between the inter-repeat loop of the CTPR and the Keap1-binding peptide. The grafting process led to the discovery of a new Keap1-binding peptide, Ac-LDPETGELL-NH_2,_ with low nanomolar affinity for Keap1, highlighting the potential of the repeat-protein class for application in peptide display.

## Introduction

**Fig. 1:**
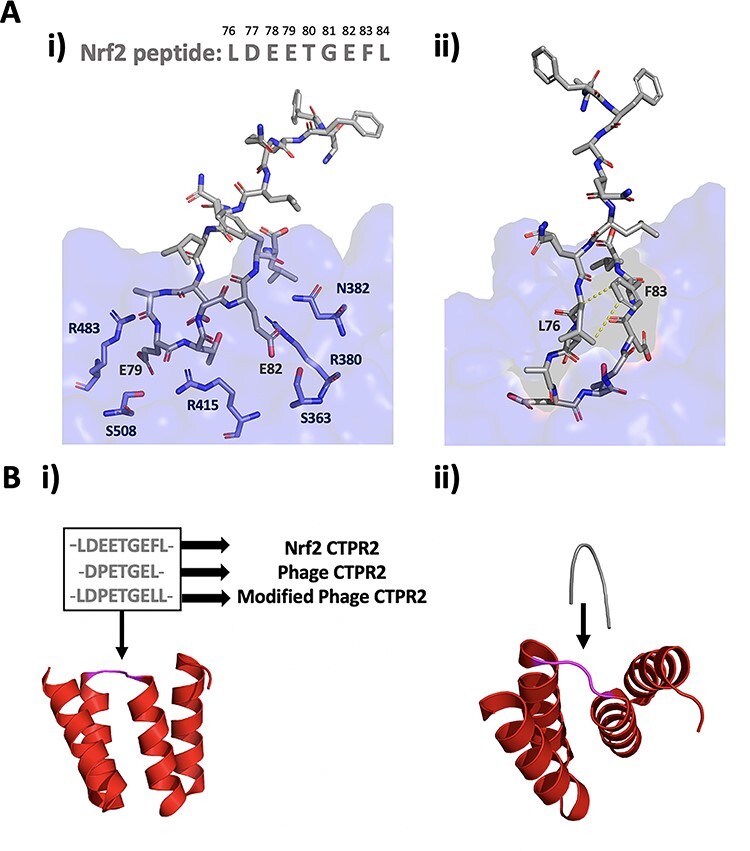
**A**: i) Schematic showing the electrostatic interactions of the Nrf2 peptide (grey) with Keap1 (blue) with numbered amino acid residues; ii) The intramolecular hydrophobic interactions of the Nrf2 peptide when bound to Keap1 (PDB ID 2FLU) (Lo *et al*. 2006). **B**: i) Nrf2, phage display-derived and modified phage display-derived peptides (grey) were grafted onto the inter-repeat loop (pink) of a CTPR2 protein (red) to create Nrf2 CTPR2, Phage CTPR2 and Modified Phage CTPR2, respectively (PDB ID 1NA0); ii) LDEETGEFL Nrf2 peptide (grey, PDB ID 1X2R) aligned with the CTPR2 protein (red, PDB ID 1NA0) (Main *et al*. 2003; Padmanabhan *et al*. 2006).

The E3 ubiquitin ligase Keap1-Cullin3 and its substrate Nrf2 function to regulate the cell’s response to electrophilic and oxidative stress (Itoh *et al*. 1997). Nrf2 is a transcription factor that upregulates a range of cytoprotective enzymes, and Nrf2 levels are tightly controlled by Keap1, which binds to and drives ubiquitination of Nrf2 leading to Nrf2 degradation (Furukawa and Xiong 2005). Targeting the Keap1-Nrf2 interaction is a therapeutic strategy in a range of diseases from the chemoprevention of cancer to the treatment Parkinson’s and Alzheimer’s disease and diabetes, and a number of small molecules are currently in clinical trials (Cuadrado *et al*. 2019; Lu *et al*. 2016; Madden and Itzhaki 2020; Wells 2015). Nrf2 has two Keap1-binding sites, the ETGE motif and the DLG motif, with each binding to a different Kelch domain of the Keap1 dimer (Canning *et al*. 2015; Horie *et al*. 2021; Tong *et al*. 2006a, b). The ETGE motif (residues 79–82) has a 100-fold higher Keap1-binding affinity compared with the DLG motif. The ETGE motif binds in a beta-turn conformation to the shallow pocket on Keap1 created by the loops connecting the beta-strands of its Kelch domain (Fig. 1A) (Lo *et al*. 2006; Padmanabhan *et al*. 2006). The Neh2 domain of Nrf2 encompassing the two motifs has been previously shown to bind to full length Keap1 with a *K*_D_ of 5–9 nM (Eggler *et al*. 2005; Lo *et al*. 2006; Tong *et al*. 2006b). The ETGE motif was found to account for the majority of the affinity, and the Neh2 domain was found to have nanomolar to low micromolar affinity when miniaturised to short ETGE peptides (Cuadrado *et al*. 2019; Hancock *et al*. 2012; Tong *et al*. 2006b).

The beta-turn of the Nrf2 ETGE motif is stabilised by three intramolecular hydrogen bonds between Asp77 and Gly81, Asp77 and Glu79, and Thr80 and Glu82 (Padmanabhan *et al*. 2006). Key interactions are made between the Glu79 of Nrf2 and Arg483, Ser508 and Arg415 of Keap1 and between Glu82 of Nrf2 and Ser363, Asn382 and Arg380 of Keap1. There has been some debate around the role of Phe83 and Leu 76 and Leu 84, which flank the ETGE motif (Fig. 1A). Hancock *et al*. (2012) have suggested that since no interactions between the Nrf2 leucine residues and Keap1 can be seen in the crystal structure, it is likely that they are instead important in enhancing intramolecular interactions within the turn conformation of the ETGE motif (Fig. 1A) (Lo *et al*. 2006). Lu *et al*. (2015) have proposed that the leucine residues form hydrophobic interactions with hydrophobic pockets of Keap1.

The large but discrete interface of the Keap1-Nrf2 interaction lends itself to inhibition using peptide-based molecules, and the crystal structure of the Kelch domain in complex with the higher affinity ETGE motif peptide has aided the design of these peptide inhibitors to date. Hancock *et al*. (2012) used a fluorescence polarisation competition assay to show that a series of ETGE motif peptides that could inhibit the Keap1-Nrf2 interaction. They also found that the ETGE peptide Ac-LDEETGEFL-OH had an order-of-magnitude lower IC_50_ than the ETGE peptide Ac-DEETGEF-OH (0.389 μM vs. 5.39 μM), reinforcing the importance of the flanking leucine residues of the ETGE motif. The authors suggest that the presence of Leu76 and Leu84 enhances the intramolecular interactions within the Nrf2 peptide, as no interactions of these residues with Keap1 can be seen in the crystal structure (Hancock *et al*. 2012). They also used phage display to identify another peptide, Ac-D**P**ETGE**L**-OH, which has a still higher affinity, with an IC_50_ of 0.115 μM. The proline (position 78) is found in the Keap1-binding region of the protein p62 and is thought to further stabilise the beta turn-forming propensity of the ETGE motif, and both this substitution and the Phe83Leu substitution highlight the importance of residues flanking the ETGE motif. A series of Nrf2-derived cyclic peptides have also been developed and *K*_**D**_ values as low as 6 nM achieved (Chen *et al*. 2019; Colarusso *et al*. 2020; Lu *et al*. 2015, 2018; Salim *et al*. 2020; Steel *et al*. 2018), although these peptides are polar and weakly cell penetrating and therefore have only low cellular activities.

Peptide grafting approaches have been previously used to impart binding functionality to protein scaffolds which serve to constrain a peptide in its bioactive conformation and improve its proteolytic stability (Stadler *et al*. 2011; Tsomaia 2015). To date, approaches have generally focused on grafting onto alpha-helices (Chin *et al*. 2001; Chin and Schepartz 2001; Montclare and Schepartz 2003; Sia and Kim 2003). There are also examples of grafting onto loops but this can be significantly more challenging to achieve by rational design, as it is difficult to predict loop conformations (Azoitei *et al*. 2012; Bonet *et al*. 2018; Der and Kuhlman 2013; Mihara *et al*. 2021; Rossmann *et al*. 2017; Sesterhenn *et al*. 2020; Sormanni *et al*. 2015). There has been some success using directed evolution and computational methods, and where the binding moiety is a short linear motif a simple cut-and-paste method may prove successful (Gilbreth and Koide 2012; Owens 2017; Rossmann *et al*. 2017; Škrlec *et al*. 2015; Stadler *et al*. 2011; Tlatli *et al*. 2013). Significant knowledge has also been gained through Complementarity-Determining Region (CDR) grafting where the CDR region is transferred from one antibody to another to improve protein stability and/or reduce immunogenicity (Ewert *et al*. 2004). A common approach is to transfer the binding region from a mouse antibody to the most closely related human antibody to reduce the immunogenicity (Jones *et al*. 1986). The factors required to increase the likelihood of successful CDR grafting are described by Ewert *et al.* as consideration of (1) residues that might be outside of the CDR region that may contribute to binding, and (2) residues capable of indirectly affecting the conformation of the antigen binding site.

Novel proteins targeting the Keap1-Nrf2 interaction have been developed through the grafting of Keap1-binding peptides onto loops of monobody, antibody and cyclotide scaffolds, as well as onto a repeat-protein scaffold by our lab (Guntas *et al*. 2016; Liu *et al*. 2017; Madden *et al*. 2019; Yin *et al*. 2021). Tetratricopeptide repeat (TPR) proteins are made up of repeating units of 34 amino acids that are composed of two antiparallel helices joined by a turn. They are widespread in nature and act as binding proteins (D'Andrea and Regan 2003). Main *et al*. (2003) optimised the stability of TPRs to create consensus-designed tetratricopeptide repeat proteins (CTPRs) through sequence alignment studies. The high stability, modular nature and absence of disulphide bonds make CTPRs ideal for protein engineering, including the introduction of new binding functions. The most well-characterised natural binding mode of TPRs is that in which the groove formed by two or three adjacent repeats interacts with a short negatively charged peptide (Allan and Ratajczak 2011; Brinker *et al*. 2002; Perez-Riba and Itzhaki 2019; Taylor *et al*. 2001). Cortajarena *et al*. (2008, 2010) were able to exploit this natural binding mode of the TPR motif to create a CTPR that bound to Hsp90 with modest mid-micromolar affinities likely due to the small interaction interface (Jackrel *et al*. 2009).

In an alternative approach, we have used the inter-repeat loop of the CTPR scaffold to engineer in binding functions. Inspection of the crystal structures of the Keap1-Nrf2 complex shows that the conformation of the beta-turn of the Nrf2 peptide is similar to that of the inter-repeat loop of the CTPRs, suggesting that it is highly suitable for grafting onto the CTPR scaffold (Fig. 1B). Following our work showing that the inter-repeat loop can be extended by up to 50 residues, we previously showed that functional peptides could be inserted into the inter-repeat loop of a CTPR to produce artificial binding proteins (Diamante *et al*. 2021; Madden *et al*. 2019). We found that a tankyrase binding-peptide could be grafted onto the inter-repeat loop of CTPRs to create a series of mono-valent and multi-valent tankyrase inhibitors. We also found that a single Nrf2 peptide could be grafted onto the inter-repeat loop of a CTPR to impart nanomolar affinity for Keap1 and that the affinity could be modulated by making mutations in the CTPR residues flanking the grafted Nrf2 sequence (Madden *et al*. 2019; Perez-Riba *et al*. 2018; Ripka *et al*. 2021). In this study, we set out to determine how grafting different Keap1-binding sequences onto the inter-repeat loop affects the stability of the CTPR scaffold and its binding affinity for Keap1 (Fig. 1B). Keap1 is a suitable choice of target for such studies because a range of peptide inhibitors with different lengths, sequences and affinities have previously been published (Hancock *et al*. 2012).

## Materials and Methods

### Protein design and molecular biology

Peptides were grafted onto the CTPR2 construct reported by Grove *et al*. (2010) with the C-terminal NN residues mutated to RS as reported by Phillips *et al*. (2012). The grafted peptide is flanked by the DPNN sequence at its N-terminus and the DPNS sequence at its C-terminus. This sequence was chosen as DPNN is the ‘native’ inter-repeat loop sequence of the CTPR and the N**→**S mutation was made to improve protein solubility (Table I).

**Table I:** Composition of grafted CTPR proteins used in this study. The peptide sequences grafted onto the CTPR loop are in black and the flanking residues in red

**CTPR**	**Loop Sequence**	**T**_**m**_ ± **SE (**°**C)**
Nrf2 CTPR2 Phage CTPR2 Modified Phage CTPR2	**DPNN** **LDEETGEFL** **DPNS** **DPNN** **DPETGEL** **DPNS** **DPNN** **LDPETGELL** **DPNS**	75.2 ± 1.1 73.0 ± 1.0 72.7 ± 1.9

**Fig. 2:**
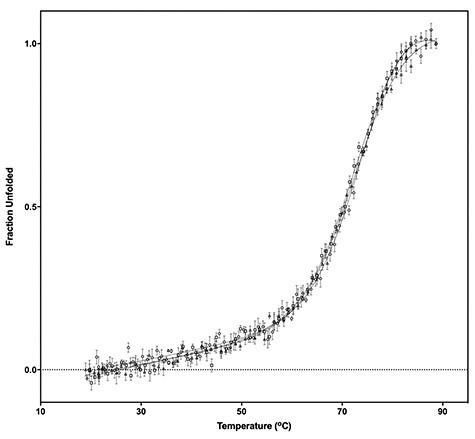
Thermal denaturation curves of Nrf2 CTPR2 (**○**), Phage CTPR2 (△) and Modified Phage CTPR2 (□) monitored by the ellipticity at 222 nm. The protein concentration was 10 μM concentration (1 mm cuvette).

The CTPR constructs were cloned using gBlock oligos (Integrated DNA technologies) into the multiple cloning site of the pRSET B vector using restriction digestion-ligation cloning with BamHI and HindIII restriction enzymes (ThermoFisher Scientific, Waltham, MA) and Quick Stick ligase (Bioline). The Keap1 Kelch domain construct (residues 321–624) was a kind donation from Alex Bullock (Structural Genomics Consortium, Oxford) with an N-terminal His-tag and a TEV cleavage site in a pNIC28-BSA4 vector.

### Protein purification

Plasmids encoding the grafted CTPRs were transformed into chemically competent *Escherichia. coli* (Lemo21 for the Keap1-binding CTPRs expression plasmids and C41 for the CTPR2n expression plasmid). Individual colonies were selected and grown in 15 ml of 2xYT media until an O.D. of 0.8 was reached (~16 h at 37°C). Cell were then induced using 0.5 mM IPTG and grown for 24 h at 20°C. Cells were pelleted and the protein extracted and subsequently purified in 50 mM Tris–HCl 150 mM NaCl as described in Perez-Riba and Itzhaki (2017). Purity was verified using mass spectrometry.

The Keap1 Kelch domain pNIC28-BSA4 expression plasmid was transformed into C41 *E. coli*. An agar plate of colonies was then resuspended in 2xYT media and grown at 37°C until an O.D. of 0.8. The cells were then induced using 0.5 mM IPTG and grown for 16 h at 20°C. The cells were subsequently pelleted at 5000 RPM, resuspended in 35 ml of 50 mM Tris–HCl pH 8 150 mM NaCl 2 mM DTT pH 8 with a Sigmafast™ protease inhibitor tablet (EDTA-free) and lysed using an Emulsiflex C-5 homogeniser. Lysates were cleared at 17 000 RPM for 45 min and incubated with 4 ml of Ni-NTA beads for 1 h at 4°C. The beads were then washed three times using 50 ml of 50 mM Tris–HCl 150 mM NaCl 2 mM DTT pH 8*,* washed with 10 ml 50 mM Tris–HCl 150 mM NaCl 30 mM imidazole 2 mM DTT pH 8 and eluted in 10 ml of 50 mM Tris–HCl 150 mM NaCl 300 mM Imidazole 2 mM DTT pH 8. The elution was filtered through a 0.22 μm syringe. Size exclusion chromatography was used as a final purification step using a HiLoad 26/60 Superdex 75 column in 50 mM Tris–HCl 150 mM NaCl 2 mM DTT pH 8.

### Synthetic peptides

Peptides were designed with N-terminal acetyl caps and C-terminal amides to better mimic the interactions seen in the grafted protein. All peptides were synthesised by Cambridge Peptides Ltd and provided at a purity of > 90%.

### Thermostability measurements

The thermal stability of the proteins was determined by monitoring protein structure at 222 nM with an Applied Photophysics Chirascan spectrophotometer. Proteins were diluted to 10 μM in 50 mM Tris 150 mM NaCl pH 8 and read in a 1 mm cuvette by subsequently heating the proteins to 94°C at a rate of 0.5°C per min, with five repeats being taken. All data were analysed using GraphPad Prism 8.0 software and curves were fitted using a sigmoidal sloppy Boltzmann equation.

### Fluorescence polarisation competition assay

A fluorescence polarisation competition assay was employed based on the assay previously reported by Hancock *et al*. (2012). Briefly, a serial dilution of CTPR was titrated into 1 nM of FITC-beta-ala-DEETGEF-OH and 182.5 nM Keap1 in 50 mM Tris–HCl 150 mM NaCl pH 8.5 and incubated for 30 min at room temperature. All experiments were carried out in 384-well black opaque Optiplate microplates in a total volume of 40 μl. The gain adjustment was set at 40 mP for the highest concentration of CTPR. The data were analysed using GraphPad Prism 8.0 and fitted using the equation reported by Wang (1995).

### Isothermal titration calorimetry assays

Proteins were buffer exchanged into 50 mM Tris–HCl 150 mM NaCl 0.5 mM TCEP pH 8 using an overnight dialysis at 4°C. All experiments were performed on a MicroCal iTC200 Microcalorimeter by titrating a 166.6 μM Keap1 solution into a 16.66 μM solution of CTPR or peptide. This was carried over 20 injections of 2 μl, with an injection duration of 0.8 s, an initial delay of 60 s, 150 s between injections, a reference power of 5 μcal/s and a stirring speed of 750 RPM. The results were subtracted from control data acquired by titrating Keap1 at the relevant concentration into buffer. All data were analysed using Origin 7.0 and subsequently fitted using a one-site binding model.

### Pull-down assay

The assay was carried out through modifying the method previously reported by Guntas *et al*. (2016) Three plates of HEK93T cells with 80% confluency were washed with PBS and subsequently lysed in 7.5 ml of 1% Triton buffer (1% Triton-X, 10% glycerol, 50 mM Tris–HCl, 150 mM NaCl, 2 mM EDTA with one Roche cOmplete mini, EDTA-free protease inhibitor cocktail tablet per 10 ml 1% Triton buffer) for 30 min on ice. DNA was sheared by passing through a 26G needle. The lysates were cleared by centrifugation at 20 000 g for 30 min at 4°C. Five batches of 50 μl Ni-NTA resin was then washed from ethanol using three 500 μl washes with 1% triton buffer (1% Triton-X, 10% glycerol, 50 mM Tris–HCl, 150 mM NaCl, 2 mM EDTA with one Roche cOmplete mini, EDTA-free protease inhibitor cocktail tablet per 10 ml 1% Triton buffer). A total of 5 ng of each CTPR and 1 ml of the HEK93T cell lysate was then incubated with each 50 μl of washed Ni-NTA batch for 3 h at 4°C. The beads were then washed three times with 500 μl 1% triton buffer and then resuspended in 20 μl SDS loading dye and boiled for 3 min. A total of 10 μl of each sample was then loaded and run on a 12% 1 mm SDS-PAGE gel with a Precision Plus Protein™ Dual Color Standards ladder (Bio-Rad). Gels were transferred to a Immobilon-P PVDF membrane (Millipore) according to the manufacturer’s instructions and blocked and blotted with Odyssey Blocking buffer, a 1 in 500 dilution of a Rabbit polyclonal anti-Keap1 primary antibody (10503–2-AP, Protein Technologies) and 1 in 1000 dilution of the IRDye®800CW Rat Anti-Rabbit secondary antibody (LI-COR) according to the LI-COR Odyssey CLx protocol and visualised using a LI-COR Odyssey CLx.

### Molecular docking

A PDB file of the LDPETGELL peptide in complex with Keap1 was created using the mutagenesis function in pyMOL and the LDEETGEFL-Keap1 crystal structure as a template (Padmanabhan *et al*. 2006). The PDB file was submitted to the FlexPepDock Server and run using 100 low-resolution simulations and 100 high-resolution simulations (London *et al*. 2011; Raveh *et al*. 2010).

**Fig. 3:**
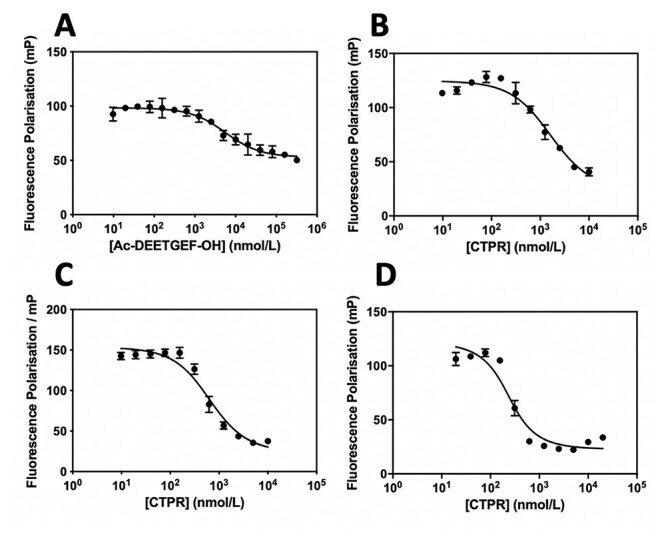
FP competition assays, **A**: Ac-DEETGEF-OH; **B**: Nrf2 CTPR2; **C**: Phage CTPR2; **D**: Modified Phage CTPR2, were titrated into a solution with a final concentration of 1 nM fluorescent tracer FITC-beta-ala-DEETGEF-OH and a 182.5 nM Keap1.

**Fig. 4:**
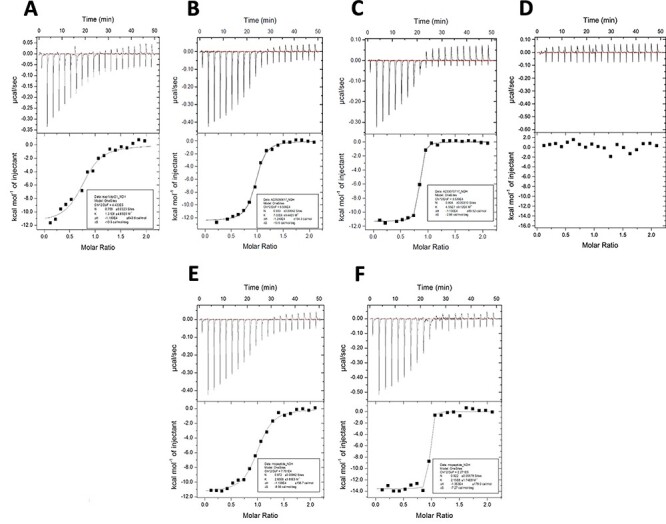
ITC measurement of the binding of the CTPR proteins and the corresponding free peptides to Keap1. A total of 166.6 μM Keap1 solution was titrated into a 16.66 μM solution of inhibitors **A**: Nrf2 CTPR2; **B**: Phage CPR2; **C**: Modified Phage CTPR2; **D**: CTPR2n; **E**: Phage display-derived peptide (Ac-DPETGEL-NH_2_); **F**: Modified phage display-derived peptide (Ac-LDPETGELL-NH_2_) over 20 injections of 2 μl, with a reference power of 5 μcal/s and a stirring speed of 750 RPM.

**Fig. 5:**
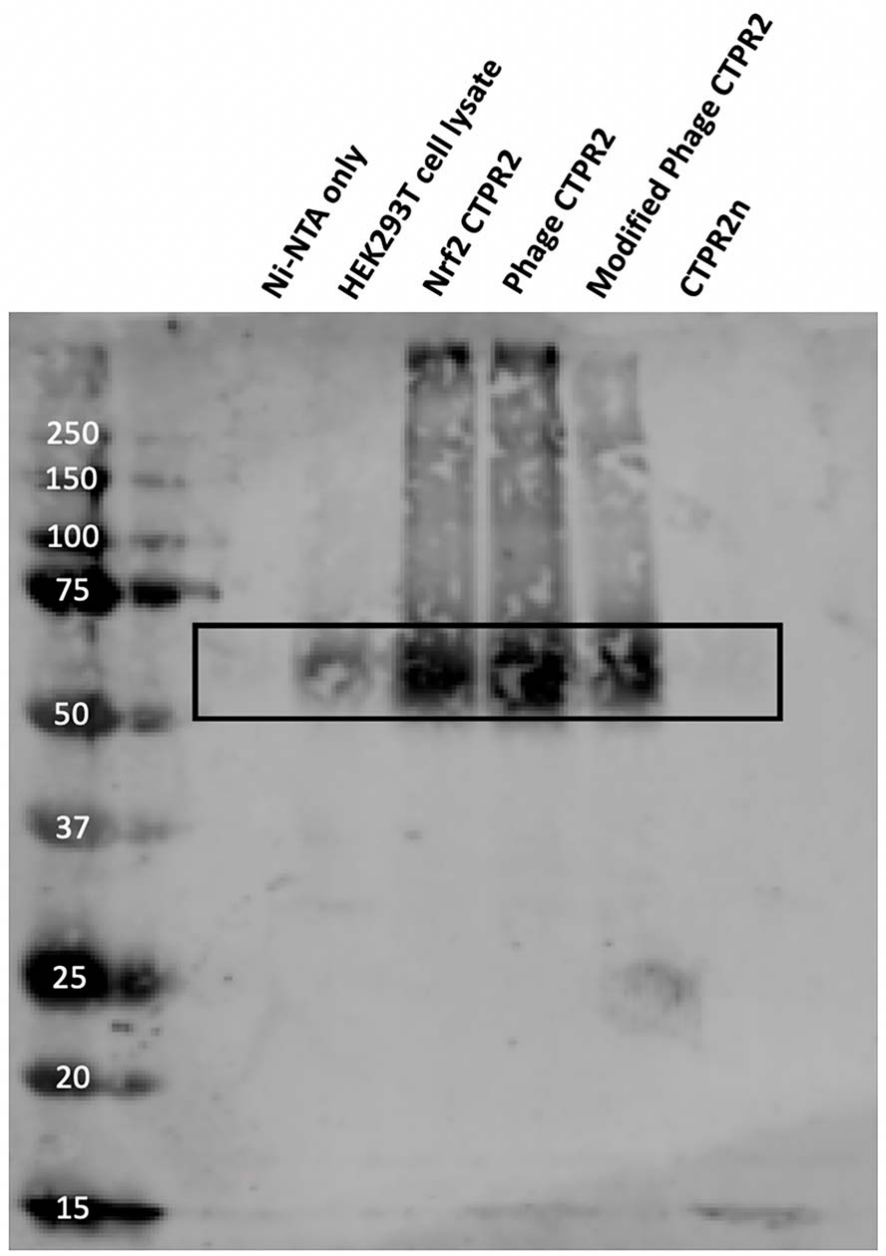
Pull-down assay of binding of CTPRs to endogenous Keap1 in HEK293T cell lysate. A total of 5 ng of each CTPR protein was incubated with 50 μl of washed Ni-NTA resin and subsequently incubated with 1 ml of cleared HEK293T cell lysates. Samples were analysed using a western blot with a 1-in-500 dilution of a Rabbit polyclonal anti-Keap1 primary antibody (10503–2-AP, Protein Technologies).

**Fig. 6:**
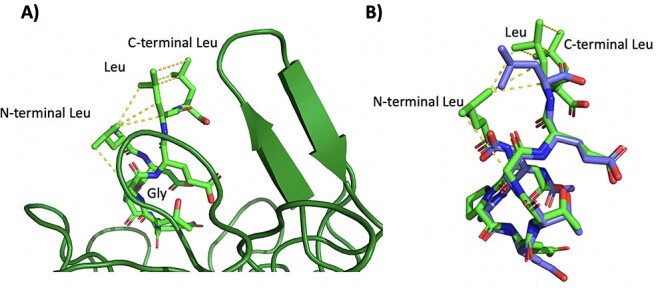
A) Docked conformation of the LDPETGELL (light green)-Kelch domain of Keap1 complex (dark green). B) Docked conformation (light green) aligned to the structure of the DPETGEL (purple) in complex with Keap1 (PDB ID 6FMQ) (Georgakopoulos *et al*. 2018).

## Results and Discussion

### CTPR design, stability and Keap1 binding

We previously reported that a single Nrf2 peptide (sequence LDEETGEFL) could be grafted onto the inter-repeat loop of a CTPR to impart nanomolar affinity for Keap1, and we investigated how changing the loop residues flanking this grafted peptide could affect protein stability and binding affinity for Keap1 (Madden *et al*. 2019). Here, we explore how the grafting of different Keap1-binding sequences affects the stability of the CTPR scaffold and its affinity for Keap1. We sought to understand how to optimise the grafting process in order maximise protein stability and affinity for the target. The fact that there are multiple Keap1-binding peptides with different sequences, lengths and affinities provides an interesting system with which to determine the effects of sequence composition and length on the functionality of the grafted peptide and on the stability of the CTPR scaffold (Hancock *et al*. 2012). Previously, we used flanking residues at both ends having the sequence DPNN, which was chosen as this is the ‘native’ CTPR loop sequence. In this study, the loop residues flanking the grafted peptides are kept constant with a DPNN sequence at the N-terminus of the grafted peptide and the DPNS sequence at its C-terminus while the grafted peptide is changed. The N**→**S mutation was used to improve protein solubility.

The Nrf2 peptide, LDEETGEFL, and the phage display-derived peptide, DPETGEL, were initially grafted onto the inter-repeat loop of a two-repeat CTPR (CTPR2) protein to make the proteins Nrf2 CTPR2 and Phage CTPR2 (Table I, Fig. 1B). We chose to graft these two peptides because they are well studied and had been previously shown to have high affinities for Keap1 in their free (unconstrained) forms (Hancock *et al*. 2012). The CTPR sequence previously reported by Grove *et al*. (2010) was used due to its high stability and solubility, and a two-repeat CTPR was used because its small size (~11.5 KDa) makes it a good minimal domain for study.

The proteins were found to have similarly high thermodynamics stabilities to those observed previously (Table I, Fig. 2). The Keap1-binding of the Nrf2 CTPR2 and Phage CTPR2 was first probed using a fluorescence polarisation (FP) competition assay used in previous studies (Hancock *et al*. 2012; Madden *et al*. 2019), and a control Keap1-binding peptide (Ac-DEETGEF-OH) with known affinity for Keap1 was included (Fig. 3A) (Hancock *et al*. 2012). The binding affinities of the Nrf2 CTPR2 and Phage CTPR2 for Keap1 was also measured using isothermal titration calorimetry (ITC), and the affinities obtained with the two types of measurements were similar (Table II, Figs 3 and 4). No binding to Keap1 was detected for a CTPR2 protein with no grafted peptide, CTPR2n (Grove *et al*. 2010). Phage CTPR2 was found to have a higher affinity than Nrf2 CTPR2 (*K*_**D**_ of 763 nM vs. 143 nM, respectively by ITC). Previously published data showed that flanking hydrophobic residues can improve the Keap1-binding affinity (Ac-LDEETGEFL-OH and Ac-DEETGEF-OH have IC_50_ values of 0.389 μM vs. 5.39 μM, respectively) (Hancock *et al*. 2012) in the native Nrf2 peptide. This result led us to explore whether extending the grafted phage display-derived DPETGEL peptide with two flanking leucine residues could also improve the binding Keap1-affinity of the CTPR protein in this context. A new construct, Modified Phage CTPR2, was therefore made using a grafted sequence LDPETGELL (Table I, Fig. 1B) (Perez-Riba and Itzhaki 2017). Modified Phage CTPR2 had a similar stability to Nrf2 CTPR2 and Phage CTPR2 and the highest affinity of any designed CTPR, with a *K*_**D**_ of 22 nM (by ITC) (Figs 2 and 4). This affinity is the highest reported for an engineered CTPR to date (Diamante *et al*. 2021; Madden *et al*. 2019). Thus, the inclusion of the two flanking leucine residues did indeed improve the binding affinity of the CTPR protein for Keap1. The CTPR scaffold was also able to accommodate both a longer 9-mer grafted peptide in the case of Nrf2 CTPR2 and Modified-Phage CTPR2, as well as a shorter 7-mer grafted peptide in Phage CTPR2.

**Table II:** Binding affinities of CTPR proteins for Keap1 measured by FP and ITC

**CTPR protein**	***K***_**i**_ ± **SE (nM)** **(FP competition assay)**	***K***_**D**_ ± **SE (nM)** **(ITC)**
Nrf2 CTPR2 Phage CTPR2 Modified Phage CTPR2 Ac-DEETGEF-OH CTPR2n	861 ± 185 301 ± 57 65.5 ± 20 3094 ± 596 n.d.	763 ± 270 143 ± 11 22.0 ± 4.4 n.d. No binding

**Table III:** Comparison of Keap1-binding affinities of the CTPR-grafted peptides to those of the free peptides measured by ITC

**Peptide**	**Peptide grafted onto CTPR scaffold** ***K***_**D**_ ± **SE (nM)**	**Free peptide** ***K***_**D**_ ± **SE (nM)**
Ac-DPETGEL-NH_2_ Ac-LDPETGELL-NH_2_	143 ± 11 22.0 ± 4.4	341 ± 45 4.65 ± 3.8

In order to probe the ability of the CTPRs to bind to endogenous Keap1, a pull-down assay was carried out using HEK293T cell lysate (Fig. 5). A band corresponding to Keap1 was observed for Nrf2 CTPR2, Phage CTPR2 and Modified Phage CTPR2, indicating that they are able to bind endogenous Keap1. No binding was observed for the control CTPR2 peptide (CTPR2n) with no grafted peptide.

### Binding of free peptides to Keap1

In order to further investigate the relationship between the binding affinities of CTPR-grafted and free peptides, the phage display-derived and modified phage display-derived peptides were synthesised and their affinities measured by ITC (Table III). The Nrf2 peptide was not measured here, as its affinity for Keap1 has already been reported (K_D_ of 138 ± 0.36 nM) (Bresciani *et al*. 2017). N-acetylated peptides with C-terminal amide groups were used to best mimic the peptide bonds either side of the grafted peptide in the CTPR inter-repeat loop. The binding affinities of the free peptides were of the same order of magnitude as those of the respective CTPR-grafted peptides. Thus, the process of grafting the peptide onto the inter-repeat loop has not significantly disrupted the binding affinities, presumably due to the good structural match between the inter-repeat loop and the beta-turn conformation of the Nrf2 peptide (Fig. 1B). This lack of disruption of peptide functionality is also likely facilitated by the robust structure of the CTPR scaffold, meaning that it can accommodate different amino acid sequences in its inter-repeat loop without comprising its stability, as shown here and in previously published work by our group (Perez-Riba *et al*. 2018). The Modified phage display-derived free peptide, Ac-LDPETGELL-NH2, has a significantly higher Keap1-binding affinity than the phage display-derived free peptide Ac-DPETGEL-NH2 (*K*_**D**_s of 4.65 nM versus 341 nM, respectively), reinforcing the important role of the flanking leucine residues. We have therefore been able to recover a similar binding affinity to that seen for the full Neh2 domain. Others have also been able to reach such affinities through the use of cyclic peptides (Chen *et al*. 2019; Colarusso *et al*. 2020; Lu *et al*. 2015, 2018).

### Molecular docking

We used molecular docking via the FlexPepDock Server in order to explore why introduction of the flanking leucine residues of the modified phage display-derived peptide, LDPETGELL, leads to the observed increase in binding affinity over the phage display-derived peptide DPETGEL (London *et al*. 2011; Raveh *et al*. 2010). Docking of the LDPETGELL peptide suggests that the leucine residues participate in intramolecular hydrophobic interactions that are absent in the DPETGEL crystal structure, mirroring the hypothesis by Hancock *et al*. (2012) as to how the flanking leucine residues in the native Nrf2 enhance the binding affinity (Georgakopoulos *et al*. 2018). Specifically, in the LDPETGELL peptide, the N-terminal leucine residue forms hydrophobic interactions with the glycine residue and penultimate leucine residue (Fig. 6). The penultimate leucine also forms hydrophobic interactions with the C-terminal leucine of the peptide, creating a large hydrophobic network within the peptide.

In summary, our study demonstrates how different Keap1-binding peptides can be grafted onto the inter-repeat loop to impart binding affinities of similar magnitudes to those of the respective free peptides in the low nanomolar range and the highest affinity CTPR for a target achieved to date. This finding is likely due to the good structural match between the inter-repeat loop of the CTPR and the turn-like conformation of Keap1-binding peptide. The identification of a new Keap1-binding peptide with low nanomolar affinity highlights the importance of the flanking leucine residues and of the mutated residues discovered through phage display, in driving high affinity for Keap1 (Hancock *et al*. 2012). This work also highlights how the CTPR scaffold can be readily exploited for the discovery of new peptide inhibitors and may be especially useful given that CTPRs can be produced in high yield with relative ease and at low cost.
